# 基于高通量全自动固相萃取的超高效液相色谱-串联质谱法测定人尿中16种抗生素和4种*β*-受体激动剂

**DOI:** 10.3724/SP.J.1123.2022.08025

**Published:** 2023-05-08

**Authors:** Zhenhuan LI, Xiaojian HU, Yifu LU, Linna XIE, Ying ZHU

**Affiliations:** 中国疾病预防控制中心环境与人群健康重点实验室, 中国疾病预防控制中心环境与健康相关产品安全所,北京100021; China CDC Key Laboratory of Environment and Population Health, National Institute of Environmental Health, Chinese Center for Disease Control and Prevention, Beijing 100021, China

**Keywords:** 固相萃取, 超高效液相色谱-串联质谱, 抗生素, *β*-受体激动剂, 尿液, solid-phase extraction (SPE), ultra-performance liquid chromatography-tandem mass spectrometry (UPLC-MS/MS), antibiotics, *β*-agonists, urine

## Abstract

建立了基于高通量全自动固相萃取的人体尿液中大环内酯、四环素、喹诺酮、磺胺4类16种常见抗生素及特步他林、沙丁胺醇、莱克多巴胺、克伦特罗4种*β*-受体激动剂的超高效液相色谱-串联质谱(UPLC-MS/MS)分析方法。尿液样本于室温解冻后,取1 mL,然后加入内标、200 μL乙酸铵缓冲液和20 μL *β*-葡萄糖醛酸酶,于37 ℃条件下酶解过夜。采用全自动固相萃取设备对尿液中的目标物进行提取,考察了固相萃取板、淋洗液、洗脱液种类及体积,结果显示采用Oasis Prime HLB 96孔固相萃取板,以1.5 mL 10%(v/v)甲醇水溶液作为淋洗液、2 mL甲醇作为洗脱液时,20种目标物的回收率最为理想。于45 ℃氮吹浓缩,比较了不同氮吹条件(完全吹干、氮吹近干、氮吹至1 mL和洗脱液中加水作为保护剂)下目标物的回收率。结果表明,在洗脱液中加入水作为保护剂时,绝对回收率最为理想。研究优化了色谱分离条件,结果显示,以HSS T_3_(100 mm×3.0 mm, 1.8 μm)作为分析柱,0.1%(v/v)甲酸水溶液-0.1%甲酸(v/v)乙腈溶液作为流动相,以0.3 mL/min流速梯度洗脱时,分离效果最好。比较了不同比例甲醇水溶液及初始流动相作为进样溶剂时的出峰情况,发现在30%(v/v)甲醇水溶液里目标物的峰形和信噪比最为理想。该方法标准曲线拟合度良好(相关系数>0.997),方法检出限为0.02~0.12 ng/mL,定量限为0.06~0.41 ng/mL。在0.25、2.5和12.5 ng/mL加标水平下,回收率为81.7%~120.0%(除四环素外),日内精密度和日间精密度(*n*=6)分别为1.1%~11.0%和1.2%~13.0%。基质效应考察结果表明,采取同位素内标校正后所有目标物均为弱基质效应。为考察该方法的正确度,采用BCR-503(含沙丁胺醇和克伦特罗)及内部质控样进行实验,结果显示沙丁胺醇和克伦特罗的测定值均在参考范围内,20种目标物在两种浓度内部质控样的7次测定浓度平均值范围分别为0.44~0.59 ng/mL(0.5 ng/mL)和1.72~2.16 ng/mL(2.0 ng/mL)。该方法采用自动化样品前处理设备结合96孔固相萃取板,有效提高了检测效率,具有操作简单、灵敏度高、回收率好、基质效应弱等优点,可满足人体尿液中16种抗生素和4种*β*-受体激动剂的同时测定需求。该研究为人体尿液中抗生素和*β*-受体激动剂的监测、暴露特征及健康风险评估提供了方法支撑。

## Abstract (en)

An analytical method combining high-throughput automatic solid-phase extraction with ultra-performance liquid chromatography-tandem mass spectrometry (UPLC-MS/MS) was developed to determine 16 antibiotics (macrolides, tetracyclines, quinolones, and sulfonamides) and 4 *β*-agonists (terbutaline, salbutamol, ractopamine, and clenbuterol) in human urine samples. After thawing at room temperature, 1 mL of urine was sampled and the internal standard was added, followed by the addition of 200 μL ammonium acetate buffer and 20 μL *β*-glucuronidase, and the mixture was incubated at 37 ℃ overnight. Automatic solid-phase extraction was used to extract the target compounds from the urine samples, and the recoveries were compared using different solid-phase extraction 96-well plates (PRiME MCX, Sep-Pak C_18_, PRiME HLB), types and volumes of rinse solutions and eluents. Satisfactory recoveries of the 20 target compounds were obtained using the Oasis PRiME HLB 96-well plate, with 1.5 mL 10% (v/v) methanol aqueous solution and 2.0 mL methanol as the rinse solution and eluent, respectively. The eluent was concentrated under nitrogen gas at 45 ℃, and the recoveries of the target compounds were compared under different conditions (completely or almost dry, drying to 1 mL, and adding water as a protective agent), and the recovery rate was optimal when water was added as a protective agent.

In this study, two types of analytical columns (ACQUITY BEH C_18_ and ACQUITY HSS T_3_) and different gradient elution procedures and mobile phases were compared. The optimal chromatographic effect was realized using an HSS T_3_ column (100 mm×3.0 mm, 1.8 μm) and 0.1% (v/v) formic acid aqueous solution-0.1% (v/v) formic acid in acetonitrile as the mobile phase in gradient elution at a flow rate of 0.3 mL/min. Comparing the peaks observed using different proportions of methanol aqueous solution and the initial mobile phase as the injection solvent revealed that 30% (v/v) methanol aqueous solution was the optimal solution in terms of peak shape and signal-to-noise ratio. MS was conducted using positive electrospray ionization (ESI^+^) in multiple reaction monitoring (MRM) mode, and the MS parameters were optimized, including the curtain (CUR) and collision gases (CAD). The standard curve obtained using this method exhibited a good linearity (correlation coefficient>0.997), and the respective limits of detection and quantification were 0.02-0.12 ng/mL and 0.06-0.41 ng/mL. At spiked levels of 0.25, 2.5, and 12.5 ng/mL, the recoveries were in the range of 81.7%-120.0% (except that of tetracycline), the intra- and inter-day RSDs (*n*=6) were 1.1%-11.0% and 1.2%-13.0%, respectively. Azithromycin, trimethoprim, terbutaline, salbutamol, ractopamine, and clenbuterol displayed moderate matrix effects, but all targets exhibited weak matrix effects after correction using the isotope internal standard. To evaluate the accuracy of this method, BCR-503 (containing salbutamol and clenbuterol) and internal quality control samples were used and the concentrations of salbutamol and clenbuterol were within the reference ranges. Additionally, the mean concentrations of the 20 target compounds of two different internal quality control samples after 7 measurements were in the ranges of 0.44-0.59 ng/mL (0.5 ng/mL) and 1.72-2.16 ng/mL (2.0 ng/mL), respectively, which were satisfactory. In this study, the analytical method employed automatic sample pretreatment with a 96-well solid-phase extraction plate, and the detection efficiency was considerably improved. This method displays the advantages of simple operation, ideal recovery, a high sensitivity and weak matrix effect, which satisfies the requirements for the simultaneous determination of 16 antibiotics and 4 *β*-agonists in human urine samples. This study provides a crucial method for use in monitoring antibiotics and *β*-agonists in human urine and studying their exposure characteristics and health risks.

抗生素因在治疗与预防感染性疾病和促进生长发育方面效果显著,被广泛应用于临床治疗、农业生产和养殖业等领域中。*β*-受体激动剂在临床上主要用于治疗哮喘、慢性阻塞性肺疾病(chronic obstructive pulmonary disease, COPD)等呼吸系统疾病,由于具有减少脂肪积累、促进蛋白质生成和提高饲料转化率的作用,曾被添加到动物饲料中以提高牲畜瘦肉率^[1]^。目前,我国已禁止硫酸特布他林、莱克多巴胺、(硫酸)沙丁胺醇和盐酸克仑特罗等在饲料和动物饮用水中的使用^[2]^。抗生素和*β*-受体激动剂通过临床用药、食物摄入或环境暴露等途径进入人体后,主要经尿液排出体外^[3⇓-5]^。近年来,其造成的人体健康问题受到普遍关注。已有人群流行病学研究显示,抗生素和*β*-受体激动剂的暴露与多种健康风险密切相关,如耐药性^[6]^、肠道菌群失调^[7]^、肥胖/超重^[8]^、心动过速^[9]^。因此,建立生物样本中多种类抗生素和*β*-受体激动剂的分析方法,了解其人群负荷水平,科学管理抗生素和*β*-受体激动剂的使用,在降低生态环境和人群健康风险等方面具有重要价值。

目前,在食品^[10,11]^、地表水^[12,13]^、土壤^[14]^、底泥^[15]^等非生物样本中抗生素和*β*-受体激动剂的分析方法研究较多,在人体生物样本中的研究相对较少。生物样本基质复杂,且抗生素和*β*-受体激动剂的负荷水平多为痕量水平,因此高效的前处理方法和高灵敏度的检测技术成为同时检测多种目标物的关键。已有报道的尿液中抗生素和*β*-受体激动剂的前处理方法主要包括固相萃取(SPE)、液液萃取、蛋白沉淀、固相分散萃取和固相微萃取等,其中SPE是目前最常用的前处理方法。检测技术主要包括微生物分析、免疫分析、高效液相色谱法(HPLC)和液相色谱-串联质谱法(LC-MS/MS)等,其中LC-MS/MS因具有灵敏度高、特异性强、分析速度快等优点,在当前检测中应用广泛。但是,已有研究多关注一种或一类目标物,同时检测尿液中抗生素和*β*-受体激动剂的分析方法鲜有报道,仅查到一篇文献同时分析了磺胺类抗生素、*β*-受体激动剂和类固醇^[16]^,该方法以非靶向筛查为主要目的,检出限较高(1~10 μg/mL),难以满足普通人群体内痕量水平目标物的定量分析需求。鉴于此,本研究建立了基于高通量全自动固相萃取的尿液中4类16种常见抗生素(大环内酯类、四环素类、喹诺酮类、磺胺类)及4种*β*-受体激动剂(特步他林、沙丁胺醇、莱克多巴胺、克伦特罗)的高效液相色谱-串联质谱(UPLC-MS/MS)检测方法。该方法操作简便,分析效率高,灵敏度高,重现性好,适合大批量人群样品的快速定量检测,具有较高的实际应用价值。

## 1 实验部分

### 1.1 仪器、材料与试剂

I Class型超高效液相色谱仪(美国Waters公司); Qtrap 6500 plus三重四极杆质谱仪(美国AB Sciex公司); 2K-15型离心机(美国Sigma公司);恒温水浴摇床(中国莱伯泰科仪器股份有限公司); 96孔氮吹仪(中国杭州米欧仪器有限公司); AP250D十万分之一电子天平(美国OHAUS公司); IQ7005纯水机(美国Milli-Q公司); 96孔固相萃取装置、Oasis Prime HLB 96孔固相萃取板、Oasis Prime MCX 96孔固相萃取板、Sep-Pak C_18_ 96孔固相萃取板与2 mL收集板(美国Waters公司); Extrahera^TM^ HV-5000大容量自动化样品制备工作站(瑞典Biotage公司)。

甲醇和乙腈(MS级,德国Merck公司);甲酸、乙酸和乙酸铵(MS级,美国Thermo Fisher公司); *β*-葡萄糖醛酸酶(>100000 U/mL,美国Sigma公司);有证参考物质BCR-503(欧洲标准局);标准品及同位素内标均购自天津阿尔塔科技有限公司,详细信息见表1。

**表1:** 20种标准品及8种内标的信息

No.	Compound	Abbreviation	Mass concentration/ Purity	Solvent
1	azithromycin (阿奇霉素)	AZI	1000 μg/mL	methanol
2	clarithromycin (克拉霉素)	CLA	1000 μg/mL	methanol
3	roxithromycin (罗红霉素)	ROX	1000 μg/mL	methanol
4	tetracycline (四环素)	TC	>96.3%	-
5	doxycycline (强力霉素)	DC	>92.4%	-
6	oxytetracycline (土霉素)	OTC	>93.8%	-
7	ciprofloxacin (环丙沙星)	CIP	1000 μg/mL	methanol
8	ofloxacin (氧氟沙星)	OFL	1000 μg/mL	methanol-dimethyl sulfoxide (1∶1, v/v)
9	enrofloxacin (恩诺沙星)	ENR	1000 μg/mL	methanol-dimethyl sulfoxide (1∶1, v/v)
10	pefloxacin (培氟沙星)	PEL	1000 μg/mL	methanol
11	difloxacin (二氟沙星)	DIF	100 μg/mL	methanol
12	norfloxacin (诺氟沙星)	NOR	100 μg/mL	methanol
13	sulfamethazine (磺胺二甲嘧啶)	SMZ	1000 μg/mL	methanol
14	sulfamethoxazole (磺胺甲噁唑)	SMX	1000 μg/mL	acetonitrile
15	sulfadiazine (磺胺嘧啶)	SDZ	1000 μg/mL	methanol-dimethyl sulfoxide (4∶1, v/v)
16	trimethoprim (甲氧苄啶)	TEP	1000 μg/mL	acetonitrile
17	terbutaline (特布他林)	TER	100 μg/mL	methanol
18	salbutamol (沙丁胺醇)	SAL	100 μg/mL	methanol
19	ractopamine (莱克多巴胺)	RAC	100 μg/mL	methanol
20	clenbuterol (克伦特罗)	CLE	100 μg/mL	methanol
21	azithromycin-D_3_ (阿奇霉素-D_3_)	AZI-D_3_	100 μg/mL	methanol
22	doxycycline-D_3_ (强力霉素-D_3_)	DC-D_3_	> 95.6%	-
23	ofloxacin-D_3_ (氧氟沙星-D_3_)	OFL-D_3_	100 μg/mL	methanol
24	sulfamethoxazole-^13^C_6_ (磺胺甲噁唑-^13^C_6_)	SMX-^13^C_6_	100 μg/mL	acetonitrile
25	terbutaline-D_9_ (特步他林-D_9_)	TER-D_9_	100 μg/mL	methanol
26	salbutamol-D_3_ (沙丁胺醇-D_3_)	SAL-D_3_	100 μg/mL	methanol
27	ractopamine-D_6_ (莱克多巴胺-D_6_)	RAC-D_6_	100 μg/mL	methanol
28	clenbuterol-D_9_ (克伦特罗-D_9_)	CLE-D_9_	100 μg/mL	methanol

-: solid.

### 1.2 溶液配制

#### 1.2.1 乙酸铵缓冲溶液

称取1.93 g乙酸铵,加入纯水溶解,转移至25 mL容量瓶,混匀。加入乙酸,调节pH为5.0,配制成1 mol/L乙酸铵缓冲溶液。

#### 1.2.2 标准储备溶液

将目标物标准品及同位素内标均用甲醇配制成质量浓度为10 μg/mL的单标储备溶液,置于-20 ℃冰箱保存。准确移取一定体积的标准品及同位素内标的单标储备溶液,用甲醇定容至10 mL,配制成质量浓度均为4.00 μg/mL的混合标准储备溶液和混合同位素内标储备溶液,置于-20 ℃冰箱保存。

#### 1.2.3 标准系列工作溶液配制

以30%(v/v)甲醇水溶液为溶剂,将4.00 μg/mL的混合标准储备溶液逐级稀释,配制标准系列工作溶液,使目标物的最终质量浓度分别为0.1、0.5、1.0、2.5、5.0、10.0、15.0、20.0、30.0 ng/mL,内标质量浓度均为24.0 ng/mL。

### 1.3 样品前处理

将冻存的尿液样本取出,放置至室温。用涡旋混匀器混匀尿液样本,准确移取1.00 mL于96孔收集板中,依次加入相当于4.8 ng的混合同位素内标,20 μL *β*-葡萄糖醛酸酶,200 μL乙酸铵缓冲溶液,于37 ℃酶解过夜。取出样品溶液冷却至室温,待进行固相萃取。

前处理过程在大容量自动化样品制备工作站中进行。依次加入1 mL甲醇和1 mL纯水对Prime HLB 96孔板进行活化、平衡。将样品溶液(约1.2 mL)分两次全部转移至96孔固相萃取板中,梯度加压模式下过柱。用0.75 mL 10%(v/v)甲醇水溶液快速淋洗,重复一次,然后用1 mL甲醇缓慢洗脱,重复一次,收集洗脱液。洗脱液中加入140 μL纯水,45 ℃条件下氮吹至140 μL,然后加入60 μL甲醇。以4000 r/min离心15 min,转移上清液至液相色谱进样小瓶中,待测。

### 1.4 分析条件

#### 1.4.1 质谱条件

离子源:电喷雾离子源(ESI);扫描模式:正电离;检测方式:多反应监测(MRM);离子源温度: 400 ℃;喷雾电压(IS): 5500 V;碰撞气(CAD): High;气帘气(CUR): 200 kPa;雾化气: 200 kPa;辅助气: 350 kPa。

#### 1.4.2 色谱条件

色谱柱:ACQUITY UPLC HSS T_3_色谱柱(100 mm×3.0 mm, 1.8 μm);流动相:A相为0.1%(v/v)甲酸水溶液,B相为0.1%(v/v)甲酸乙腈溶液;流速:0.3 mL/min;柱温箱温度:40 ℃;样品室温度:10 ℃;进样量:5 μL。流动相洗脱梯度见表2。

**表2:** 梯度洗脱程序

Time/min	*φ*(A)/%	*φ*(B)/%
Initial	97.0	3.0
1.0	97.0	3.0
2.0	80.0	20.0
7.0	40.0	60.0
8.5	5.0	95.0
11.0	5.0	95.0
11.5	97.0	3.0
15.0	97.0	3.0

A: 0.1% (v/v) formic acid aqueous solution; B: 0.1% (v/v) formic acid in acetonitrile.

## 2 结果与讨论

### 2.1 质谱条件优化

依次对单个目标物进行质谱参数优化。首先采用母离子扫描模式(Q1 scan),确定目标物的分子离子峰,再采用子离子扫描模式(product ion scan),确定子离子峰;选取2个丰度高、干扰少的子离子分别作为定量离子和定性离子;对目标物的离子对进行碰撞电压(CE)和去簇电压(DP)的优化。分别对CUR、CAD、离子源温度、雾化气和辅助气进行优化,根据目标物的信号强度,选择最优条件。CUR为300 kPa时,大环内酯类抗生素无响应,而改为200 kPa时,均有响应,且该条件下其他目标物的信噪比(*S/N*)变化不大,最终选择CUR为200 kPa(图1a)。比较CAD为Low、Medium和High时,各目标物的信号强度,结果显示,CAD为High时,多数目标物响应最好(图1b)。改变离子源温度(400、500 ℃)、雾化气(200、350 kPa)和辅助气(350、380 kPa)等参数,目标物信号强度变化较小,为降低分析成本,选择离子源温度为400 ℃, 雾化气为200 kPa, 辅助气为350 kPa。最终确定的20种目标物及内标的主要质谱参数见表3。

**图1:**
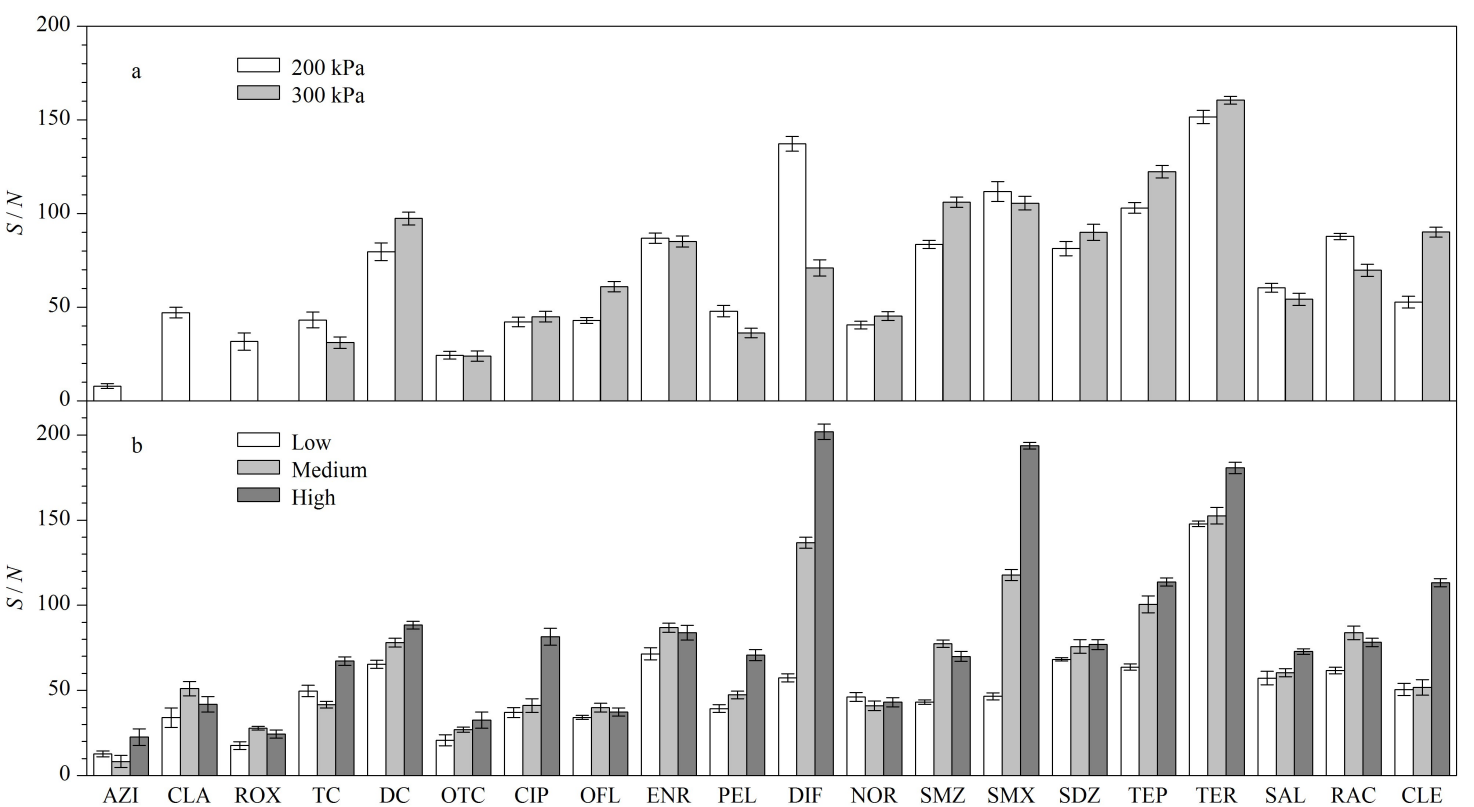
不同(a)气帘气及(b)碰撞气条件下20种目标物的信噪比(*n*=4)

**表3:** 20种目标物及8种内标的质谱参数

No.	Compound	Precursor/ (*m/z*)	Product/ (*m/z*)	CE/eV	DP/V
1	AZI	749.7	591.3^*^	44	60
			573.5	48	
2	CLA	748.6	158.2^*^	34	10
			590.3	25	
3	ROX	837.6	679.3^*^	30	30
			158.1	43	
4	TC	445.2	154.0^*^	34	20
			410.0	27	
5	DC	445.2	428.1^*^	25	45
			410.1	35	
6	OTC	461.1	426.0^*^	26	35
			443.1	18	
7	CIP	332.2	288.2^*^	22	120
			231.0	26	
8	OFL	362.2	318.0^*^	24	120
			261.0	39	
9	ENR	360.2	316.3^*^	28	35
			342.0	31	
10	PEL	334.1	233.0^*^	32	130
			290.1	26	
11	DIF	400.2	356.1^*^	25	125
			299.0	35	
12	NOR	320.2	276.0^*^	20	130
			233.1	33	
13	SMZ	278.9	186.2^*^	31	25
			156.2	31	
14	SMX	254.1	91.8^*^	24	45
			108.1	31	
15	SDZ	251.1	156.1^*^	20	63
			92.0	28	
16	TEP	291.3	230.1^*^	36	50
			261.2	30	
17	TER	226.1	152.1^*^	20	70
			107.1	35	
18	SAL	240.1	148.0^*^	19	60
			166.0	17	
19	RAC	302.3	121.1^*^	30	40
			136.0	26	
20	CLE	277.1	203.0^*^	20	30
			168.0	34	
21	AZI-D_3_	752.6	594.4	46	30
22	DC-D_3_	448.3	431.0	26	30
23	OFL-D_3_	365.2	261.1	35	30
24	SMX-^13^C_6_	260.1	162.0	25	30
25	TER-D_9_	235.1	153.1	28	25
26	SAL-D_3_	243.2	151.0	20	30
27	RAC-D_6_	308.3	168.0	21	24
28	CLE-D_9_	286.1	204.1	22	40

* Quantitative ion; CE: collision energy; DP: declustering potential.

### 2.2 色谱条件优化

#### 2.2.1 色谱柱选择

考察了ACQUITY UPLC BEH C_18_色谱柱(100 mm×2.1 mm, 1.7 μm)^[17,18]^和HSS ${T_{3}}^{\left[19,20\right]}$ 两种色谱柱。结果表明,相同流动相条件(0.1%(v/v)甲酸水溶液-0.1%(v/v)甲酸乙腈溶液)下,使用HSS T_3_色谱柱时,磺胺类(图2a)和大环内酯类(图2b)目标物的*S/N*更高,约为BEH C_18_色谱柱条件下的1.4~2.5倍。可能的原因是:与BEH C_18_色谱柱相比,HSS T_3_色谱柱采用三键C_18_烷基键合,配基密度低,对极性化合物的保留效果更好。鉴于多数抗生素属于极性化合物,最终选用HSS T_3_色谱柱。

**图2:**
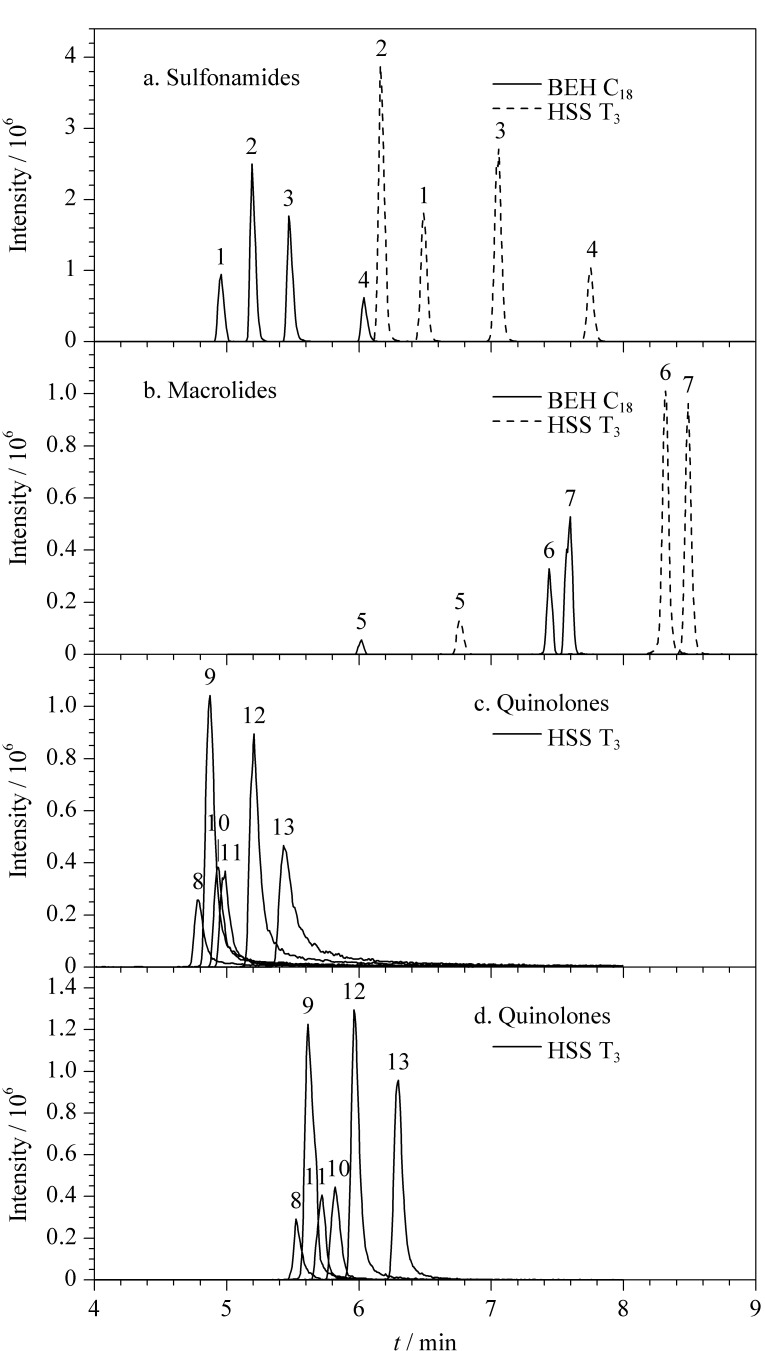
采用不同色谱柱及流动相时目标物的提取离子色谱图

#### 2.2.2 流动相优化

通过查阅文献,发现在抗生素或*β*-受体激动剂分析中,常用的水相为纯水或酸性水溶液,有机相为甲醇、乙腈或其酸性溶液。因此,本研究采用ACQUITY UPLC HSS T3色谱柱,比较了水-甲醇、水-乙腈、0.1%(v/v)甲酸水溶液-0.1%(v/v)甲酸甲醇溶液和0.1%(v/v)甲酸水溶液-0.1%(v/v)甲酸乙腈溶液等不同流动相条件下各目标物的*S/N*和分离效果。结果发现,水-乙腈和水-甲醇作为流动相、不加甲酸时,大部分物质易产生拖尾现象,其中喹诺酮类抗生素最为严重(图2c)。为提高正离子质子化,改善色谱峰形,在流动相中加入0.1%(v/v)甲酸。在0.1%(v/v)甲酸水溶液-0.1%(v/v)甲酸甲醇溶液和0.1%(v/v)甲酸水溶液-0.1%(v/v)甲酸乙腈溶液两种流动相体系下,各物质峰形较好(图2d)。主要是因为加酸后,H^+^与目标物RO^-^结合,抑制目标物水解,改善拖尾现象;同时又为正离子的形成提供了必需的质子来源,从而提高其离子化效率。考虑到甲醇黏性大于乙腈,采用UPLC进行分离时,柱压偏高,随着色谱柱进样次数的增多,容易出现超压现象,最终选择乙腈作为有机相。

#### 2.2.3 梯度洗脱条件优化

以0.1%(v/v)甲酸水溶液-0.1%(v/v)甲酸乙腈溶液作为流动相,采用梯度洗脱程序优化分离效果。当初始流动相中有机相比例为20%(维持2 min)、 10 min内增加到90%时,各目标物出峰时间过早,且在3.0~3.7 min内密集出峰,分离度差。当初始有机相比例降低为10%(维持2 min),并在10 min内增加到90%时,大部分目标物的出峰时间可以延后约3 min,分离度较好,但特步他林和沙丁胺醇仍出峰很快,容易受到溶剂效应的影响。通过进一步优化,当初始流动相中有机相比例降低至3%,并在11 min内通过梯度洗脱程序分段缓慢增加有机相比例至95%时,特步他林和沙丁胺醇的出峰时间可延后至5 min左右,其他目标物在5.5~8.2 min以内出峰完全,分离度良好。优化后的梯度洗脱程序见表2。

#### 2.2.4 进样溶剂优化

采用纯甲醇为溶剂进样时,由于溶剂效应会出现色谱峰拖尾或分叉等现象,因此,需降低溶剂中的有机相比例。本研究比较了不同体积分数(90%~10%)的甲醇水溶液和初始流动相为进样溶剂时,各目标物的峰形和*S/N*。结果发现,随着有机相的减少,各目标物峰形得到改善,当甲醇比例降低到40%时,均无拖尾等现象,峰形较好。信号响应方面,进样溶剂主要影响大环内酯类抗生素,该类物质微溶于水,易溶于有机溶剂,使用水相比例较高的初始流动相或甲醇水溶液作为进样溶剂时信号响应会降低,而有机相比例的增加有利于提高其*S/N*^[21]^。综合考虑其他目标物的信号响应,最终选择30%(v/v)甲醇水溶液作为进样溶剂。20种目标物的MRM色谱图见图3。

**图3:**
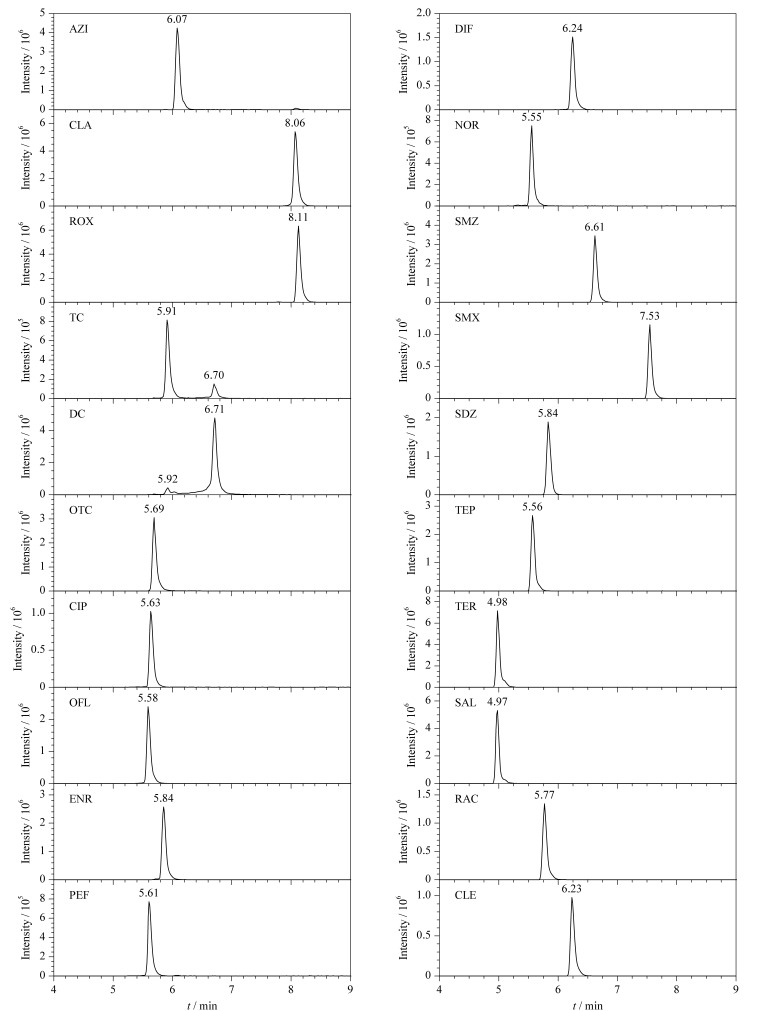
MRM模式下20种目标物(20 ng/mL)的色谱图

### 2.3 前处理条件优化

#### 2.3.1 固相萃取柱的选择

采用全自动固相萃取设备进行前处理。根据目标物的极性、p*K*_a_、相对分子质量,结合文献查阅结果,比较了Prime MCX、Sep-Pak C_18_、Prime HLB等3种96孔固相萃取板对尿液中目标物的萃取效果。图4表明,采用Prime MCX柱时,仅磺胺类抗生素的萃取效果较为理想,这可能是由于磺胺类抗生素结构中含有多个氨基基团,在酸性条件下容易形成阳离子,利用阳离子交换柱进行净化和富集是较为理想的方法^[22]^。采用Sep-Pak C_18_柱时,四环素类和喹诺酮类物质萃取效果差,而Prime HLB柱应用范围广,适用于极性、非极性和弱极性的化合物,对于本研究关注的20种目标物均有较好的净化富集效果,最终选择Prime HLB 96孔萃取板提取样品。

**图4:**
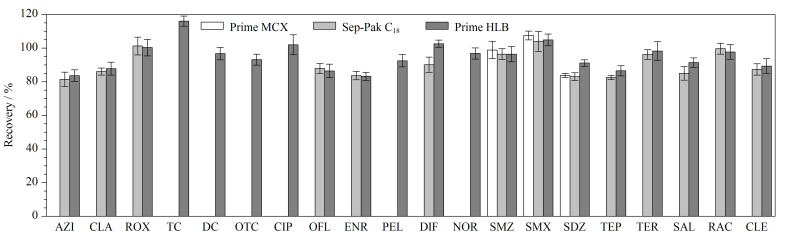
固相萃取柱对目标物回收率的影响(*n*=4)

#### 2.3.2 淋洗条件优化

本研究比较了不同体积分数(0%~30%)的甲醇水溶液作为淋洗液时目标物的淋洗损失情况。损失率以淋洗液中目标物内标峰面积与相同浓度条件下未经处理的标准溶液中内标峰面积的比值表示,比值越大损失越多。结果发现,随着甲醇比例的增加,淋洗液中各目标物损失增多,*β*-受体激动剂类物质尤为明显。以沙丁胺醇为例,30%甲醇水溶液为淋洗液时,损失率为48.5%, 20%甲醇水溶液中为36.5%, 10%甲醇水溶液中为8.5%,纯水淋洗几乎无损失。为减少目标物的损失并尽可能去除其他有机杂质,最终选择10%甲醇水溶液作为淋洗液。经进一步优化淋洗液的使用量(0.5~2.5 mL),发现使用1.5 mL淋洗液时,各目标物绝对回收率高,杂峰干扰少。因此,本研究选择1.5 mL 10%(v/v)甲醇水溶液作为淋洗液。

#### 2.3.3 洗脱条件优化

本研究比较了甲醇、乙腈、丙酮、乙酸乙酯、二氯甲烷等不同有机溶剂对目标物洗脱效果的影响,并以洗脱液中内标峰面积与相同浓度条件下未经处理的标准溶液中内标峰面积比值表示,比值越接近100%,说明洗脱效果越好。结果显示,采用二氯甲烷作为洗脱液时,磺胺类物质(磺胺二甲嘧啶、磺胺甲噁唑、磺胺嘧啶)的洗脱效果较好,但其他物质未被洗脱;采用乙酸乙酯时,仅可洗脱四环素类和磺胺类物质;采用甲醇、乙腈或丙酮时,可洗脱所有目标物,且对于多数目标物,甲醇和乙腈的洗脱效果普遍优于丙酮。以阿奇霉素为例,以甲醇作为洗脱液时的比值为89%,而采用乙腈和丙酮时,分别为39%和42%。综合考虑,选择甲醇作为洗脱液。另外,考察了洗脱液的使用量(0.5~3.0 mL)对洗脱效果的影响。结果表明,当洗脱液的使用量达到2.0 mL后,各目标物的绝对回收率无明显提高,说明2.0 mL甲醇能够满足目标物的洗脱需求,为节约溶剂,减少氮吹时间,本研究选择以2.0 mL甲醇作为洗脱液。

#### 2.3.4 氮吹条件优化

比较了在45 ℃条件下,完全吹干、氮吹至近干、氮吹至1 mL和洗脱液中加纯水作为保护剂等不同氮吹条件对目标物绝对回收率的影响。结果发现,氮吹至近干时,各目标物的绝对回收率为完全吹干的1.1~6.9倍,说明氮吹至近干可在一定程度上减少目标物的损失,但与氮吹至1 mL相比,磺胺类物质的绝对回收率偏低,这可能是因为磺胺类物质易在氮吹过程中损失。考虑到随着氮吹时间的增加,部分目标物也会随有机相的挥发而损失,本研究尝试了在洗脱液中加入纯水作为保护剂的方式,并且为保证最终进样溶剂比例与2.2.4节所述一致,先于洗脱液中加入140 μL纯水作为保护剂后氮吹至140 μL,再加60 μL甲醇作为最终进样溶液。结果显示,该条件下目标物绝对回收率明显优于氮吹至近干的条件,且与氮吹至1 mL相比,能够提高各目标物的富集倍数,有利于降低方法的检出限,更适合尿液中痕量目标物的分析要求。因此,本研究选择先加入纯水作为保护剂后氮吹的方式。

### 2.4 基质效应评价

由于无法获得不含20种目标物的实际尿液,因此采用人工尿液作为基质,配制质量浓度分别为0.1、0.5、1.0、2.5、5.0、10.0、15.0、20.0、30.0 ng/mL的基质标准系列工作溶液,经前处理,与标准系列工作溶液一同上机测定,并按文献方法^[23]^对绝对基质效应进行评估,用基质效应(matrix effects, ME)的绝对值表示。结果显示,阿奇霉素、甲氧苄啶、特步他林、沙丁胺醇、莱克多巴胺和克伦特罗的|ME|为23.0%~37.0%,为中等基质效应,需采取补偿措施。其余目标物的|ME|为0.6%~18.0%,为弱基质效应。在此基础上,进一步评估内标对基质效应的校正效果。结果表明,表现出中等基质效应的6种目标物经过校正后的|ME|为4.5%~17.0%,基质效应得到校正。鉴于*β*-受体激动剂类物质有中等基质效应,定量时采用一一对应内标,其他目标物依据化学性质和结构组成,选择阿奇霉素-D_3_、强力霉素-D_3_、氧氟沙星-D_3_和磺胺甲噁唑-^13^C_6_分别作为大环内酯、四环素、喹诺酮、磺胺4类抗生素的内标。

选取6份不同来源的实际尿样,经过前处理后,分别加入混合内标,计算各目标物内标峰面积的相对标准偏差(RSD),用于评估不同基质对信号响应的影响。结果显示,在不同基质中内标峰面积的RSD为3.9%~14.8%,说明信号响应稳定。为提高工作效率,定量分析时可用溶剂标准曲线代替基质工作曲线。

### 2.5 方法学评价

#### 2.5.1 方法检出限与定量限

以目标物与内标质量浓度之比为横坐标(*x*),峰面积之比为纵坐标(*y*)绘制校准曲线,内标法定量,线性方程见表4。在测量浓度范围内,所有目标物的相关系数(*r*)均大于0.997。采用美国环保署(US EPA)方法^[24]^,确定本方法的方法检出限(MDLs)和方法定量限(MQLs)。鉴于所有目标物均没有空白干扰,用不含本底的尿样预估方法定量限,并以此浓度进行加标,用7次测定结果的3倍和10倍标准偏差分别计算MDLs和MQLs。20种目标物的MDLs和MQLs分别为0.02~0.12 ng/mL和0.06~0.41 ng/mL(见表4)。

**表4:** 20种目标物的线性范围、回归方程、相关系数(*r*)、方法检出限和方法定量限

No.	Compound	Linear range/(ng/mL)	Linear equation	*r*	MDL/(ng/mL)	MQL/(ng/mL)
1	AZI	0.1-30	*y*=0.0652*x*+0.0114	0.998	0.03	0.09
2	CLA	0.1-30	*y*=0.0593*x*+0.0069	0.999	0.02	0.08
3	ROX	0.1-30	*y*=0.0674*x*+0.0014	0.999	0.03	0.09
4	TC	0.1-30	*y*=0.0252*x*+0.0008	0.999	0.03	0.08
5	DC	0.1-30	*y*=0.0592*x*+0.0043	0.999	0.05	0.12
6	OTC	0.1-30	*y*=0.1160*x*+0.0188	0.999	0.02	0.06
7	CIP	0.5-30	*y*=0.0348*x*+0.0103	0.999	0.10	0.33
8	OFL	0.1-30	*y*=0.0392*x*+0.0061	0.999	0.02	0.06
9	ENR	0.5-30	*y*=0.1050*x*+0.0046	0.999	0.07	0.24
10	PEL	0.1-30	*y*=0.0251*x*+0.0024	0.998	0.03	0.10
11	DIF	0.1-30	*y*=0.0245*x*+0.0021	0.999	0.04	0.12
12	NOR	0.5-30	*y*=0.0547*x*+0.0354	0.999	0.09	0.30
13	SMZ	0.1-30	*y*=0.1140*x*+0.0115	0.999	0.02	0.06
14	SMX	0.5-30	*y*=0.0257*x*+0.0043	0.998	0.12	0.41
15	SDZ	0.1-30	*y*=0.0430*x*+0.0064	0.997	0.03	0.09
16	TEP	0.1-30	*y*=0.0405*x*+0.0066	0.999	0.03	0.12
17	TER	0.1-30	*y*=0.0571*x*+0.0322	0.998	0.03	0.06
18	SAL	0.1-30	*y*=0.3640*x*+0.0049	0.999	0.02	0.06
19	RAC	0.5-30	*y*=0.0139*x*+0.0008	0.998	0.08	0.26
20	CLE	0.5-30	*y*=0.0473*x*+0.0066	0.999	0.08	0.25

*y*: peak area ratio of compound to internal standard; *x*: mass concentration ratio of compound to internal standard.

#### 2.5.2 回收率与精密度

采用实际尿样加标的方式,配制20种目标物的低(0.25 ng/mL)、中(2.5 ng/mL)、高(12.5 ng/mL)水平加标溶液,进行加标回收率和精密度试验。结果表明,低、中、高3个水平的加标回收率分别为83.2%~120.0%、83.1%~122.0%和81.7%~116.1%,日内精密度(*n*=6)和日间精密度(*n*=6)分别为1.1%~11.0%和1.2%~13.0%,具体见表5。本研究除四环素外,其他目标物的加标回收率为81.7%~120.0%,满足GB/T 27417-2017^[25]^要求(回收率范围为60%~120%)。四环素的加标回收率为122%,其原因可能是四环素无对应内标。另外,本方法所有目标物的精密度结果均符合GB/T 27417-2017标准对精密度的要求(低浓度下精密度小于43%)。

**表5:** 20种目标物的加标回收率、日内精密度和日间精密度(*n*=6)

No.	Compound	0.25 ng/mL				2.5 ng/mL				12.5 ng/mL				
		Recovery/%	Intra-day RSD/%	Inter-day RSD/%		Recovery/%	Intra-day RSD/%	Inter-day RSD/%		Recovery/%		Intra-day RSD/%	Inter-day RSD/%	
1	AZI	92.6	2.3	4.4		83.1	4.1	6.0		81.7		11.0	7.1	
2	CLA	88.4	2.7	5.6		92.4	1.4	4.4		91.9		7.2	7.3	
3	ROX	99.3	1.2	7.2		99.7	2.4	5.8		93.2		2.2	2.7	
4	TC	104.4	3.8	4.6		122.0	4.7	5.3		116.6		6.5	5.6	
5	DC	95.5	1.4	4.6		99.5	3.2	1.8		98.8		7.9	5.0	
6	OTC	85.8	5.3	13.0		91.1	9.4	8.1		91.9		7.3	2.8	
7	CIP	95.5	3.2	3.7		105.9	6.7	8.1		96.3		5.3	6.3	
8	OFL	83.5	4.7	6.4		89.0	2.9	2.9		86.0		6.0	7.8	
9	ENR	83.9	6.3	8.6		88.2	2.8	5.5		87.1		9.8	10.0	
10	PEL	93.3	6.2	5.3		97.6	1.6	2.7		96.0		5.1	5.2	
11	DIF	101.6	2.6	2.0		100.0	4.6	3.6		103.8		2.2	2.4	
12	NOR	109.7	5.3	6.9		94.6	3.1	2.2		99.8		9.3	5.4	
13	SMZ	99.9	1.2	1.6		105.4	2.8	2.5		103.5		6.2	6.1	
14	SMX	101.4	9.1	8.7		100.9	1.8	2.0		99.5		1.1	1.2	
15	SDZ	89.2	1.4	7.2		93.0	1.9	1.9		94.9		2.6	2.4	
16	TEP	83.2	2.9	5.2		84.0	2.1	4.7		88.6		4.7	4.6	
17	TER	102.8	5.4	5.5		103.8	7.8	5.1		111.9		2.1	2.3	
18	SAL	96.6	3.7	3.6		91.0	2.7	3.2		86.6		3.4	4.0	
19	RAC	120.0	5.8	5.6		98.1	4.8	5.7		95.6		3.1	3.6	
20	CLE	92.7	5.0	6.2		99.1	2.9	3.5		92.8		6.9	7.7	

#### 2.5.3 正确度评价

通过测定含沙丁胺醇和克伦特罗的BCR-503质控样品,评估方法的正确度。结果表明,沙丁胺醇和克伦特罗的测定结果分别为2.8~3.1 ng/mL (RSD: 4.2%,*n*=4)和2.1~2.2 ng/mL (RSD: 3.8%,*n*=4),均在给定的参考值范围1.7~3.2 ng/mL和2.1~2.9 ng/mL之内,说明测定结果正确。

因大部分目标物没有对应的有证参考物质,故本研究同时设置质量浓度为0.50 ng/mL和2.00 ng/mL的内部质控样品(internal quality control, IQC)来进一步评估方法的正确度,且每个浓度进行7次平行测定。在0.50 ng/mL的内部质控样品中,20种目标物测定浓度平均值的范围为0.44~0.59 ng/mL, RSD为4.4%~9.6%;在2.00 ng/mL的内部质控样品中,20种目标物测定浓度平均值的范围为1.72~2.16 ng/mL, RSD为3.6%~9.4%,见图5。表明该方法正确度较好,结果准确。

**图5:**
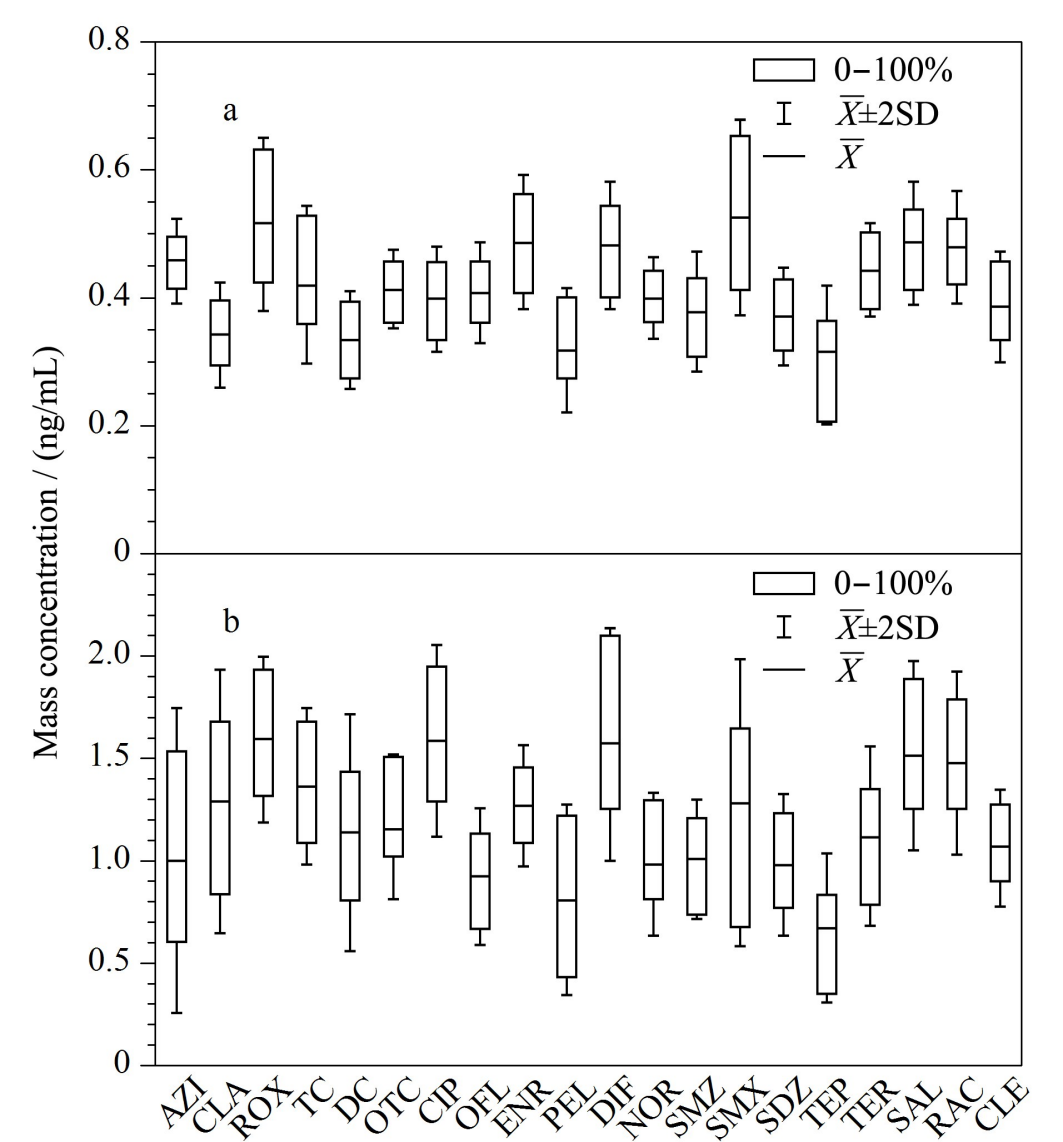
内部质控样的正确度评价结果(*n*=7)

## 3 结论

本研究建立了基于高通量全自动固相萃取的UPLC-MS/MS方法用于测定人体尿液中16种抗生素和4种*β*-受体激动剂。相较于已有的抗生素检测方法,本研究增加了4种*β*-受体激动剂,实现了人体尿液中多种抗生素和*β*-受体激动剂的同时测定;采用全自动固相萃取设备,通过优化前处理条件,提高了工作效率,节省了前处理试剂用量;通过优化仪器条件,改善了色谱峰形,增强了目标物的信号响应。本方法操作简单,灵敏度高,基质效应弱,回收率理想,满足人体尿液中多种痕量抗生素和*β*-受体激动剂的高通量分析需求,可为人体尿液中抗生素和*β*-受体激动剂的暴露研究提供方法支撑。
